# Spatial scaling in bed‐site selection by roe deer fawns: Implications for mitigating neonatal mortality during mowing

**DOI:** 10.1002/ece3.10729

**Published:** 2023-11-28

**Authors:** Sophie Baur, Johanna Kauffert, A. J. Mark Hewison, Sophie Reinermann, Andreas König, Annette Menzel, Wibke Peters

**Affiliations:** ^1^ Bavarian State Institute of Forestry, Research Unit Wildlife Biology and Management Freising Germany; ^2^ Professorship of Ecoclimatology, TUM School of Life Sciences Technical University of Munich Freising Germany; ^3^ Université de Toulouse, INRAE, CEFS Castanet‐Tolosan France; ^4^ LTSER ZA PYRénées GARonne Auzeville Tolosane France; ^5^ Department of Remote Sensing, Institute of Geography and Geology University of Würzburg Wuerzburg Germany; ^6^ Wildlife Biology and Management Unit, TUM School of Life Sciences Technical University of Munich Freising Germany; ^7^ Institute for Advanced Study Technical University of Munich Garching Germany

**Keywords:** *Capreolus capreolus* L., forage risk trade‐off, human‐wildlife conflicts, mowing death, scale‐dependency

## Abstract

When habitat use by field‐dwelling animals coincides in space and time with agricultural practices such as spring mowing of meadows, human‐wildlife conflicts can have deadly consequences for wildlife. Roe deer (*Capreolus capreolus* L.) fawns are particularly vulnerable because they hide in meadows during the rearing phase. Thus, a better understanding of the habitat drivers of bed‐site selection is critical to mitigating fawn mortality during mowing. Here, we tease apart the among‐field (presumably driven by maternal behaviour) and within‐field (driven by fawn behaviour) components of bed‐site selection of roe deer during the spring mowing season. We collected over 600 fawn bed sites across an environmentally diverse study region. At the among‐field scale, we implemented a used versus available design and employed a two‐part statistical model (GAMLSS) to identify habitat characteristics that were linked to either fawn presence (vs. absence) or abundance on a given field. At the within‐field scale, we compared habitat characteristics at fawn bed‐sites with paired random sites using a conditional logistic regression model. At the among‐field scale, fawns were more likely to be present, and were more abundant, in fields within more diverse, rural landscapes, with nearby woodland. Surprisingly, fawns were more often present in fields that were near roads and had lower vegetation productivity. At the within‐field scale, however, fawns preferred bed‐sites which were further from both roads and woodland, but that provided the best visual cover to minimise predation risk. Our findings revealed substantial and novel scale‐dependent differences in the drivers of habitat selection of mothers and fawns, which, together, determine the precise locations of bed‐sites between and within meadows. These results may aid wildlife managers in identifying areas where there is a high probability of encountering a roe deer fawn so as to initiate targeted searches prior to mowing and, ultimately, mitigate fawn mowing mortality.

## Graphical abstract

Grassland mowing is a major cause of mortality for roe deer fawns and a text‐book example for human‐wildlife conflicts due to the lack of information on factors that characterise bed‐sites. We differentiated between the maternal and neonatal behavioural components to explain the scale‐dependent biological drivers of bed‐site selection of roe deer with an innovative approach.

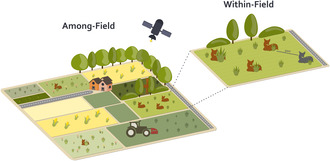

## 1 INTRODUCTION

The behavioural process of choosing a given resource, or habitat patch, amongst the array of available resources within a given environment is defined as habitat selection. This process links the behaviour of an individual to the resources it requires during different life stages to finance growth and reproduction. Indeed, habitat selection is expected to impact fitness (Gaillard et al., 2010), for example through a close link to lifetime reproductive success (McLoughlin et al., 2007; Regan et al., 2016). Maternal behaviour and, in particular, habitat selection during parturition and lactation can have a crucial influence on the growth (Théoret‐Gosselin et al., 2015) and survival (Kjellander et al., 2012; McLoughlin et al., 2007) of her offspring. In fact, mothers must select a habitat that simultaneously meets their own very high energetic demands during lactation (Oftedal, 1985), while minimising the risk of predation to themselves, but especially to their highly vulnerable offspring (Bongi et al., 2008; Ciuti et al., 2006, 2009; Panzacchi et al., 2010). These constraints are particularly acute for income breeders, such as roe deer (*Capreolus capreolus* L.), that rely on current intake to offset the increased energetic requirements associated with maternal care (Andersen et al., 2000). To meet these requirements, mothers may be obliged to adjust their space use, resulting in life history stage‐dependent variations in foraging, social and movement behaviour (e.g. Malagnino et al., 2021; Ozoga et al., 1982). The diverse activities of humans are increasingly overlapping in space and time with the habitat requirements of wildlife, particularly in densely populated or intensively exploited landscapes. For species that thrive in human‐dominated landscapes, such as roe deer, both the negative and positive influences of human presence can be substantial (Bonnot et al., 2013).

With approximately 15 million individuals (IUCN Red List, 2015) and covering a geographical range of 7.2 million km^2^ (Burbaite & Csányi, 2009), roe deer is the most numerous and widespread deer species in Europe (Apollonio et al., 2010; Linnell & Andersen, 1998; Linnell, Duncan & Andersen, 1998; Linnell, Wahlström & Gaillard, 1998). While it was historically considered a forest‐dwelling species, roe deer today also successfully use agricultural landscapes (Hewison et al., 1998). These provide both rich feeding habitat for the mother (Hewison et al., 2009) and bed‐sites with sufficient concealment for fawns (Christen et al., 2018; Panzacchi et al., 2010). In particular, during spring, meadows (i.e. grasslands) are attractive habitats for birth and rearing activities (Linnell et al., 2004). Roe deer fawns are hiders (e.g. Lent, 1974), and the hiding phase is sustained over the first 1–2 months of life, which coincides with spring mowing (Linnell, 1994).

Because this strategy against natural predators (e.g. foxes) is to remain motionless in hiding (Aanes & Andersen, 1996), many young fawns fall victim to mowing machinery during grass, silage and hay production in spring. Mowing is a major cause of fawn mortality in cultivated landscapes (Jarnemo, 2002). Dead fawn carcasses that are incorporated into silage and hay can potentially contaminate livestock with botulinum toxins (Moeller Jr. & Puschner, 2007). Therefore, great efforts are undertaken to reduce fawn mowing deaths in agricultural landscapes, for example by scanning fields with aerial thermographic cameras on unmanned aerial vehicles (UAVs) prior to mowing (Cukor et al., 2019; Israel, 2011). However, mowing activity often occurs simultaneously at a local scale in relation to plant phenology and windows of suitable weather conditions. Furthermore, fawn births are highly synchronised within a given population, with 80% occurring in less than one month (Gaillard et al., 1993), so not all fields can be systematically and efficiently searched. Hence, detailed knowledge on favoured bed‐sites can assist wildlife managers in a targeted search, which is the underlying motivation of our study.

Research on bed‐site selection is scarce and has primarily focused on the selection of specific habitat types (Christen et al., 2018), motivated by understanding natural mortality risk (Panzacchi et al., 2010; Van Moorter et al., 2009). However, to date, there is a lack of information on how roe deer choose bed sites in cultivated landscapes where fawns are particularly vulnerable to human mowing activity. Thus, we aimed to identify the habitat drivers of fawn bed‐site selection at both the among‐ and within‐field scales, approximating the maternal behavioural component in terms of her choice of where to give birth and rear her fawns (among‐field scale) and the neonatal behavioural component in terms of the fawns' decision on where to hide (within‐field scale), respectively. Although their habitat use is constrained by the space use of their mother, fawns appear to choose the exact location of their bed‐sites following each bout of maternal care (e.g. Lent, 1974). This may minimise the presence of maternal olfactive cues at the bed‐site (Lent, 1974), which might otherwise attract predators. In addition, because of their size‐to‐volume ratio, fawns are susceptible to hypothermia, limiting the availability of suitable bed‐sites (Linnell et al., 1995; Van Moorter et al., 2009). Building upon these multi‐scale considerations, we herein test the following hypotheses:

1. Among‐field scale: bed‐site selection of roe deer fawns is constrained by the home range behaviour of their mothers, that is the mother's decision on where to give birth and care for her young.
  - Preference for locations that provide high forage quality that offset the mother's high energetic demands during lactation for this income breeding ungulate (high NDVI, high portion of crops and forest, low portion of mown fields; Borowik et al., 2013; Oftedal, 1985; Pettorelli et al., 2011).
  - Preference for locations with a higher degree of spatial heterogeneity that can provide cover and forage for the mother within a short distance of her fawn's bed‐site [higher land‐cover diversity (Shannon index) and close distance to wooded patches; Christen et al., 2018; Tufto et al., 1996; Van Moorter et al., 2009].
  - Preference for locations with a low level of human disturbance to decrease perceived risk (further from roads and low presence of man‐made structures; Bonnot et al., 2013).
2. Within‐field scale: the exact location of the fawns bed‐site within a habitat patch is determined by the fawn's choice on where to hide after suckling.
  - Preference for bed‐sites that minimise predation risk by foxes and human disturbance in terms of good cover (visibility, vegetation height, shorter distances to wooded patches and further distances to roads; Bonnot et al., 2013; Christen et al., 2018; Panzacchi et al., 2009; Tufto et al., 1996).
  - Preference for bed‐sites that provide optimal thermoregulation given by warm and dry locations (Site Severity Index and by high and dense vegetation; Kurt, 1978; Van Moorter et al., 2009).

## 2 MATERIALS AND METHODS

### 2.1 Study area

**FIGURE 1:**
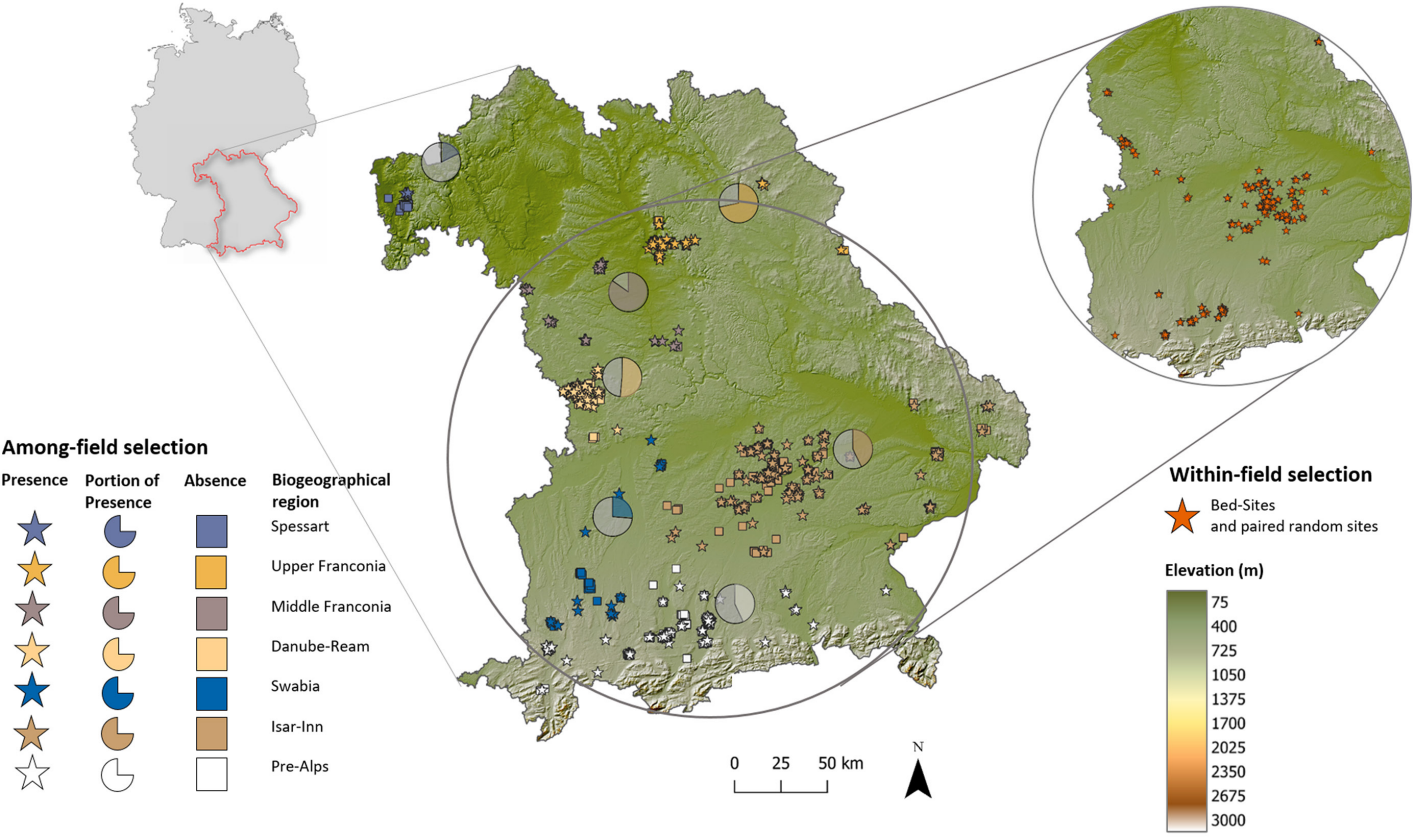
Locations of fields surveyed during spring in 2020 and 2021 for the presence (indicated by stars) or absence (indicated by squares) of fawns in Bavaria, Germany, grouped by biogeographical regions [modified from Ssymank (1994)] (DEM: SRTM 30 m resolution).

The study was conducted in Bavaria, the most south‐eastern federal state of Germany (Figure 1), which is characterised by strong climate gradients, high landscape heterogeneity and intense land use. About 49% of its area is used for agriculture and, thereof, nearly one‐third is grassland with an average field size of 1.5 ha (Bayerisches Staatsministerium für Ernährung, Landwirtschaft und Forsten, 2020). The first mowing for silage usually takes place between 10 May and 20 May and is then followed by up to three more cuts that coincide with the hiding phase of fawns (mean parturition date in Bavaria: 15 May, standard deviation 10 days; Kauffert et al., 2023). The fields used for hay production are, on average, mown for the first time between 30 May and 14 June (average in 1992–2017; Deutscher Wetterdienst, 2022).

### 2.2 Among‐field selection

#### 2.2.1 Data collection

Data were collected from 21 April to 25 June in 2020 and 2021 in agricultural areas, mainly just prior to mowing events. Besides fawn observations by our own search teams mainly in Danube‐Ream, Pre‐Alps and Isar‐Inn (see section 2.3 and Figure 1), we used citizen science data that were recorded on a website (Wildtierportal Bayern; https://www.wildtierportal.bayern.de/ wildtierrettungsstrategien) and an online survey (LimeSurvey Project Team, 2022). Both tools allowed drone pilots, hunters and farmers to report both the location of fawns and fields where no fawns were observed during pre‐mowing search operations. Meadows were mainly searched by UAVs with thermal infrared cameras across all three data sources. If at least one fawn bed‐site was found in a given field, we recorded it as a presence point. If no fawn was detected within a meadow, we considered it an ‘absence field’. Although most fawn search methods, for example with UAVs, yield very high detection rates (Cukor et al., 2019), we cannot be sure that all fawns were found. However, we here assume that we have found all the fawns.

**FIGURE 2:**
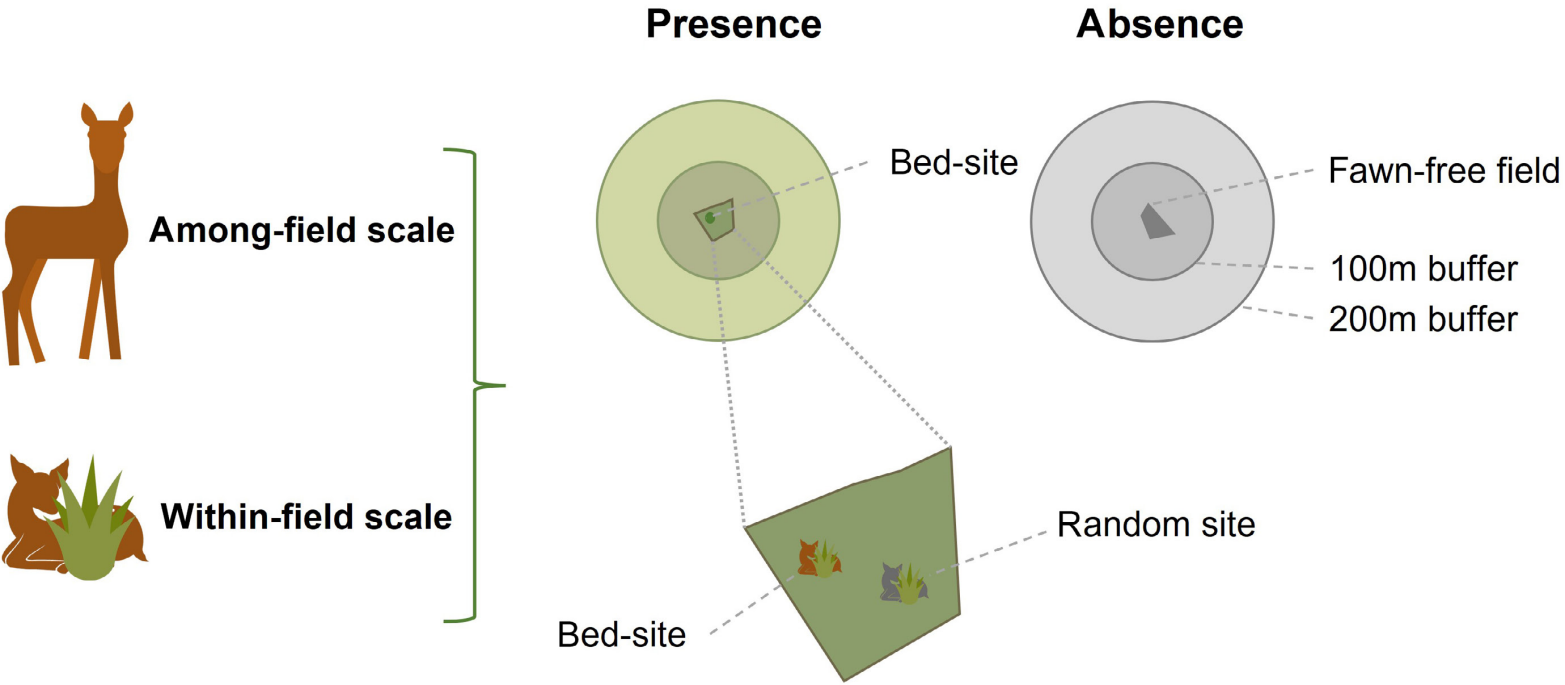
Study design for the analysis of bed‐site selection in relation to habitat characteristics at the among‐field and within‐field scale.

We obtained data for 602 fawns in 410 fields (min: 1, mean: 1.46, max: 7 fawns/field) and 469 fawn‐free fields (total fields: *n* = 879) for our analyses. To make the exact fawn location (point) and the fawn‐free fields (area) comparable, we calculated the number of fawns per ha per field. We used land‐parcel borders from the InVeKoS‐Database (Bayerisches Staatsministerium für Ernährung, Landwirtschaft und Forsten, 2020) to delimit field polygons (Figure 2). For each fawn location, we characterised the habitat within a circular buffer with a radius of 100 and 200 m (usually maintained distance to fawn 50–150 m; Espmark, 1969; Linnell & Andersen, 1998; Linnell, Duncan & Andersen, 1998; Linnell, Wahlström & Gaillard, 1998) and home range sizes during lactation (S. Baur *unpublished data*) around each bed‐site and averaged the habitat descriptors per field and day of search. For the fawn‐free fields, we randomly selected five points within the field, applied circular buffers (100 and 200 m) and averaged habitat descriptors per field. During preliminary analysis using univariate models, we investigated the explanatory power of each habitat descriptor using the aforementioned buffers. In most cases, the buffer of 100 m radius was the most informative (lowest AIC value), thus, all habitat descriptors were generated with a 100 m buffer centred on the fawn's location or on the five randomly assigned points in fawn‐free fields (see Table S1).

#### 2.2.2 Habitat descriptors

Land‐cover: We used the European Space Agency WorldCover 10 m 2020 product (Zanaga et al., 2021) to characterise landscape structure. We accessed the product via Google Earth's Engine Python API (Gorelick et al., 2017). Land‐cover composition was determined by calculating the proportion or presence of the following classes within each buffer: cropland, forest, urban and grassland. Secondly, we calculated the Shannon index to describe landscape heterogeneity within each buffer (Jung, 2016; Shannon, 1948) with SciPy (1.6.1) (Jones et al., 2001) in Python (3.8.2). Lastly, the minimum distance to nearby wooded patches (forests or hedges) and roads was calculated in metres (log‐transformed) based on the federal topographic information system (Bayerische Vermessungsverwaltung, 2020).

Forage availability: As a proxy for forage availability, we used the Normalised Difference Vegetation Index (NDVI) from satellite remote sensing data (Pettorelli et al., 2011). Due to the fine spatial scale of our analysis, we used Sentinel‐2 Level‐2A data {Bottom Of Atmosphere, spatial resolution: 10 m [Band 4 (Red) and 8 (NIR)], temporal resolution: 5 days} data and retrieved NDVI statistics (mean, standard deviation, minimum, maximum) within each buffer for mid‐April (corresponding to the start of the birth season) and for a date close to the day of fawn search from Google Earth Engine's Python API (Gorelick et al., 2017). For the latter, we selected images corresponding to the day of search with less than 20% cloud cover. If images were too cloudy, we gradually increased the temporal scope around the day of the search (limit: 12 days in each direction). We applied cloud and shadow masks to both products based on the SCL product of Sen2Cor (Louis et al., 2016).

Mown fields: We calculated the proportion of mown fields within each buffer on the day of the search (excluding the field of interest, since it was, by definition, not mown by the time of search). Grassland mowing events were detected based on Sentinel‐2 time series data from the years 2020 and 2021, as laid out in Reinermann et al. (2022).

#### 2.2.3 Statistical analyses

To evaluate the preferences of roe deer females on where to give birth and care for their fawn at the among‐field scale, we used the number of fawns per ha as the response variable and the aforementioned habitat descriptors as explanatory variables. Due to potential regional differences in environmental context (roe deer density, hunting pressure and predation risk) and diversity of data collection efforts and sources (see secton 2.2.1), we included the biogeographical region [seven regions, after Ssymank (1994) see Figure 1] as a random effect. We assume that the aforementioned factors vary little within a region. Additionally, to account for within‐season variation in the proportion of fawns born at the time of the search, we included the time period of the search as a three‐modality random effect [(1) search before 15.05; (2) search between 15.05 and 31.05; (3) search later than 31.05] as roe deer birth dates are expected to be normally distributed (Gaillard et al., 1993; Linnell & Andersen, 1998).

Subsequently, we conducted a Spearman's Rank Correlation analysis to evaluate multicollinearity among habitat descriptors. We grouped covariates according to our hypotheses and, when covariates within a given group were correlated (correlation |*r*| ≥ 0.60), we retained the covariate that was most strongly related to the response variable [lowest Akaike's Information Criterion (AIC)] with an univariate modelling approach using GAMLSS (see below). Next, within the previously selected variables, we used the variance inflation factor (VIF) to sequentially drop variables with high collinearity (threshold: 2) (Casals et al., 2021; Zuur et al., 2010) using RStudio (Version 2022.07.1) (R Studio Team, 2020) (R‐Package: car; Fox & Weisberg, 2019).

We aimed to model the absence vs. presence of fawns within fields and, additionally, when present, their abundance to test for the attractiveness of fields. However, during explanatory analysis, we noticed zero‐inflation in our response variable [more observations of absence than presence; similar to Casals et al. (2021)], leading to unstable parameter estimates with GLMER (Gamma distribution, R‐package: lme4). Thus, we alternatively used Generalised Additive Models for Location, Scale and Shape (GAMLSS) by Rigby et al. (2005) fitted with a zero‐adjusted Gamma distribution (ZAGA) (Stasinopolulos et al., 2017). The GAMLSS allows us to model the absence vs. presence component as a binomial distribution and the abundance component as a continuous distribution. This statistical approach, with its high flexibility due to various families, outperforms the usual habitat selection studies, which often rely on separate models in a resource selection function framework only (Panzacchi et al., 2010). The GAMLSS, in contrast, allowed us to simultaneously model the abundance of the response variable within the *μ*‐component (mean), whereas the *ν*‐component (skewness) models the probability of zero (absence) with a single modelling framework (Casals et al., 2021). We log‐transformed variables describing distances to the nearest landscape features as well as the response variable, which was the number of fawns per ha per field. We performed a stepwise Generalised Akaike Information Criterion (GAIC) to select the best model.

### 2.3 Within‐field selection

#### 2.3.1 Data collection

To evaluate the bed‐site preferences of fawns in terms of habitat descriptors, trained persons (own research groups) searched for fawns in targeted meadows with UAVs or by systematically walking through the meadows. When a neonate was detected, it was aged in relation to a series of behavioural and morphological characteristics based on a combination of the approaches used in Jullien et al. (1992) and Rehnus et al. (2018). Fawns were classified into two groups: ‘<2 weeks’ (day 1–14) and ‘≥2 weeks’ (>day 14) for this analysis. For each recorded bed‐site, we surveyed a paired random site at a distance of 50 m in a randomly chosen direction (Figure 2). At this spatial scale, 322 bed‐sites (and 267 random sites) were included in the analysis.

#### 2.3.2 Habitat descriptors

At each bed‐site and each paired control, the mean vegetation height (cm) was measured at two points (at opposite edges of a 1 m^2^ plot around the site) using a standardised falling plate [modified after Rayburn and Rayburn (1998)]. The plate (acrylic plastic) was 25 cm by 25 cm, weighted 128 g with a standardised hole in the middle. It was dropped along a folding rule, 10–15 cm above the vegetation canopy, to ensure that differences in falling speed would not distort the measurements. Fawn detectability was estimated by measuring the minimum distance (cm) at which at least a part of a standardised object representing a bedded fawn was visible to a potential predator in two randomly chosen cardinal directions, 50 cm above ground level. We calculated the distance to the closest wooded patch and road (m), as described above (see section 2.2.2). To characterise thermal conditions, the Site Severity Index (SSI) was calculated according to Nielsen and Haney (1998) from a digital elevation model (10 m resolution) (Bayerische Vermessungsverwaltung, 2020). The SSI describes the intensity of solar radiation at a given site as a function of slope and aspect (Nielsen & Haney, 1998). Thus, warm‐dry (south‐west slopes, indicated by positive values) and cool‐humid locations (north‐east slopes, indicated by negative values) locations can be distinguished, which we assumed to represent favourable and non‐favourable conditions for fawns, respectively.

#### 2.3.3 Statistical analyses

We analysed bed‐site selection through a direct comparison of pairs of used (=1) and control (=0) sites using conditional logistic regression (clogit) in RStudio (Version 2022.07.1) (R‐Package: survival; R Studio Team, 2020; Therneau, 2022) within a matched case‐control framework (Hosmer & Lemeshow, 2000). As for the among‐field selection scale above, we log‐transformed variables describing the distances to the nearest wooded and patch road and tested for multicollinearity. We modelled the selection of bed‐sites with respect to paired random sites in relation to the above habitat descriptors, including an interaction term between fawn age and all variables except SSI (Van Moorter et al., 2009).

We generated models for all possible combinations of the explanatory variables. The models were then ranked using AIC adjusted for small sample sizes (AICc). We listed all models within ∆AICc_Model_ ≤ 2 units of the top candidate model (Burnham & Anderson, 2002), but considered the model with the lowest AICc as the top‐ranked model (Arnold, 2010).

## 3 RESULTS

### 3.1 Among‐field selection

The best fitting GAMLSS (lowest GAIC) supported habitat descriptors (proportion of surrounding grassland mown, proportion of crops, presence of man‐made structures, distance to nearest wooded patch, land‐cover diversity, mean NDVI in April, max NDVI, distance to nearest road) related to the probability of a fawn being present and fawn abundance (Table 1).

**TABLE 1:** Summary statistics of the best GAMLSS‐model based on GAIC describing the probability of fawn absence (*ν*) and abundance (*μ*) in relation to the habitat descriptors.

	Estimate	Std. error	*t* Value	Pr(|t|)	Signif. codes
*ν* link function: logit – Probability of no fawns (absence) on a field					
(Intercept)	1.4520	1.9136	0.759	0.4482	
Proportion of surrounding grassland mown	−0.0519	0.3323	−0.156	0.8760	
Proportion of crops	0.7364	0.5044	1.460	0.1447	
Presence of man‐made structures	1.4866	0.2172	6.843	<0.001	***
Distance to nearest wooded patch	0.2318	0.0952	2.436	0.0151	*
Land‐cover diversity	−7.9768	3.5114	−2.272	0.0234	*
Mean NDVI in April	−1.0488	0.9229	−1.136	0.2561	
Max NDVI close to day of search	−0.0374	0.3676	−0.102	0.9191	
Distance to nearest road	0.2675	0.1030	2.597	0.0096	**
*μ* link function: log – Fawn abundance on a field given there was at least one fawn					
(Intercept)	−0.7361	0.5189	−1.419	0.1564	
Proportion of surrounding grassland mown	0.4292	0.1157	3.711	<0.001	***
Presence of man‐made structures	0.1142	0.0758	1.507	0.1321	
Distance to nearest wooded patch	−0.1141	0.0257	−4.443	<0.001	***
Land‐cover diversity	2.4723	0.9973	2.479	0.0134	*
Mean NDVI in April	−0.6154	0.2659	−2.314	0.0209	*
	AIC	1231.39			
	*R* ^2^	0.35			

*Note*: The dependent variable is the log of the number of fawns per ha. Signif. codes: ***p < 0; **.001; *.01; ‘.’.05.

#### 3.1.1 Fawn presence

**FIGURE 3:**
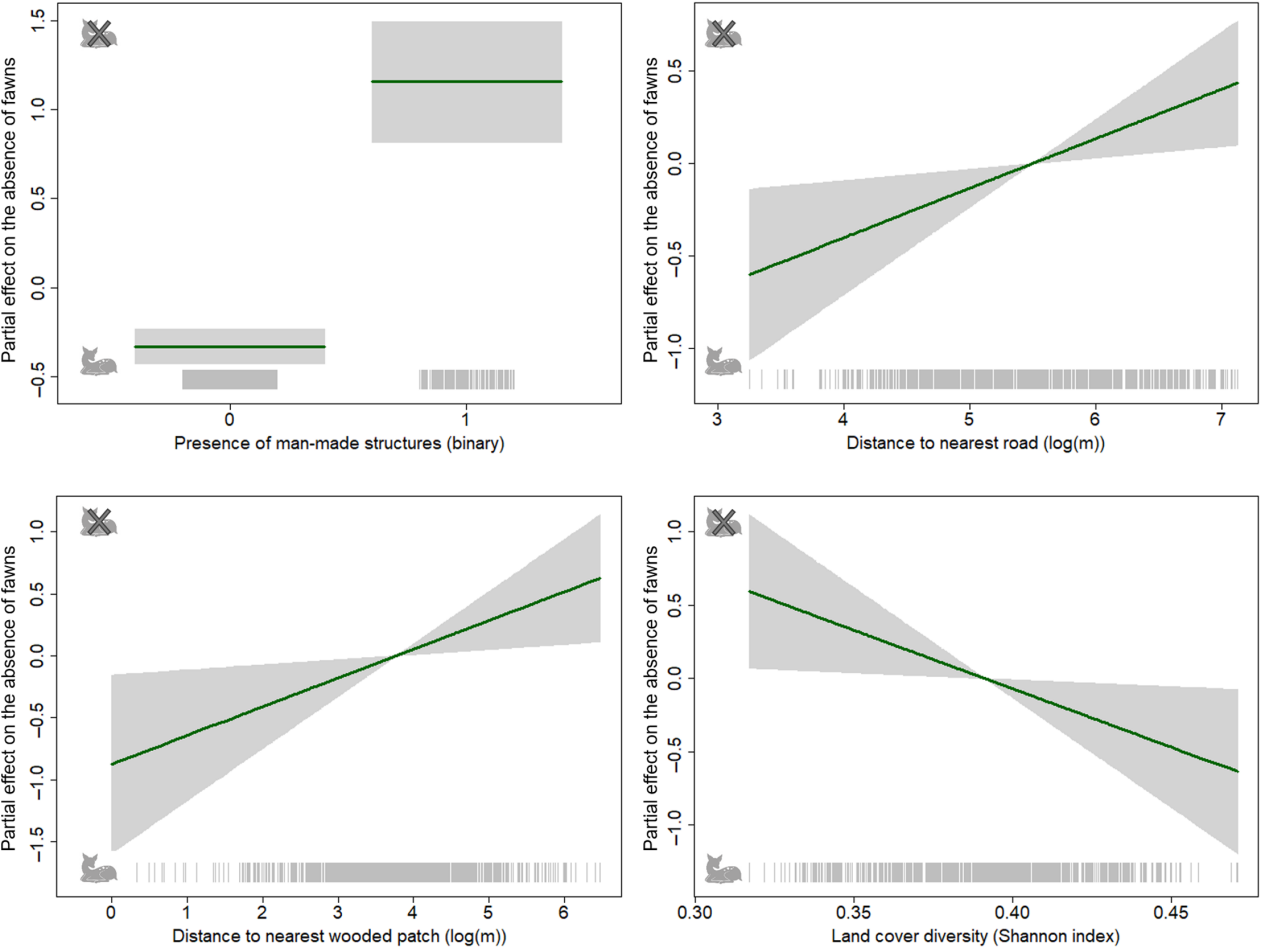
Partial regression plots for the significant explanatory variables (see Table 1) describing the absence of fawns in a field (*ν*). Shaded grey areas represent the standard errors. Positive values (crossed‐out fawn) indicate a lower chance of a fawn being present.

The *ν*‐component of the GAMLSS model indicates the probability of absence, that is the probability that no fawn was present in a field. Thus, a positive parameter estimate indicates that an increase in the value of the respective habitat descriptor decreases the chances of a fawn being present. The probability of a fawn being present was lower when there were man‐made structures within the 100 m buffer [1.487 ± 0.217 (*Estimate* ± SE)]. In contrast, the probability of a fawn being present increased as the distance to the nearest road decreased [0.268 ± 0.103 (*Estimate* ± SE)]. The probability of fawns being present was also higher as land‐cover diversity increased [−7.977 ± 3.511 (*Estimate* ± SE)] and as the distance to the nearest wooded patch decreased [0.232 ± 0.095 (*Estimate* ± SE); Table 1 and Figure 3].

#### 3.1.2 Fawn abundance

**FIGURE 4:**
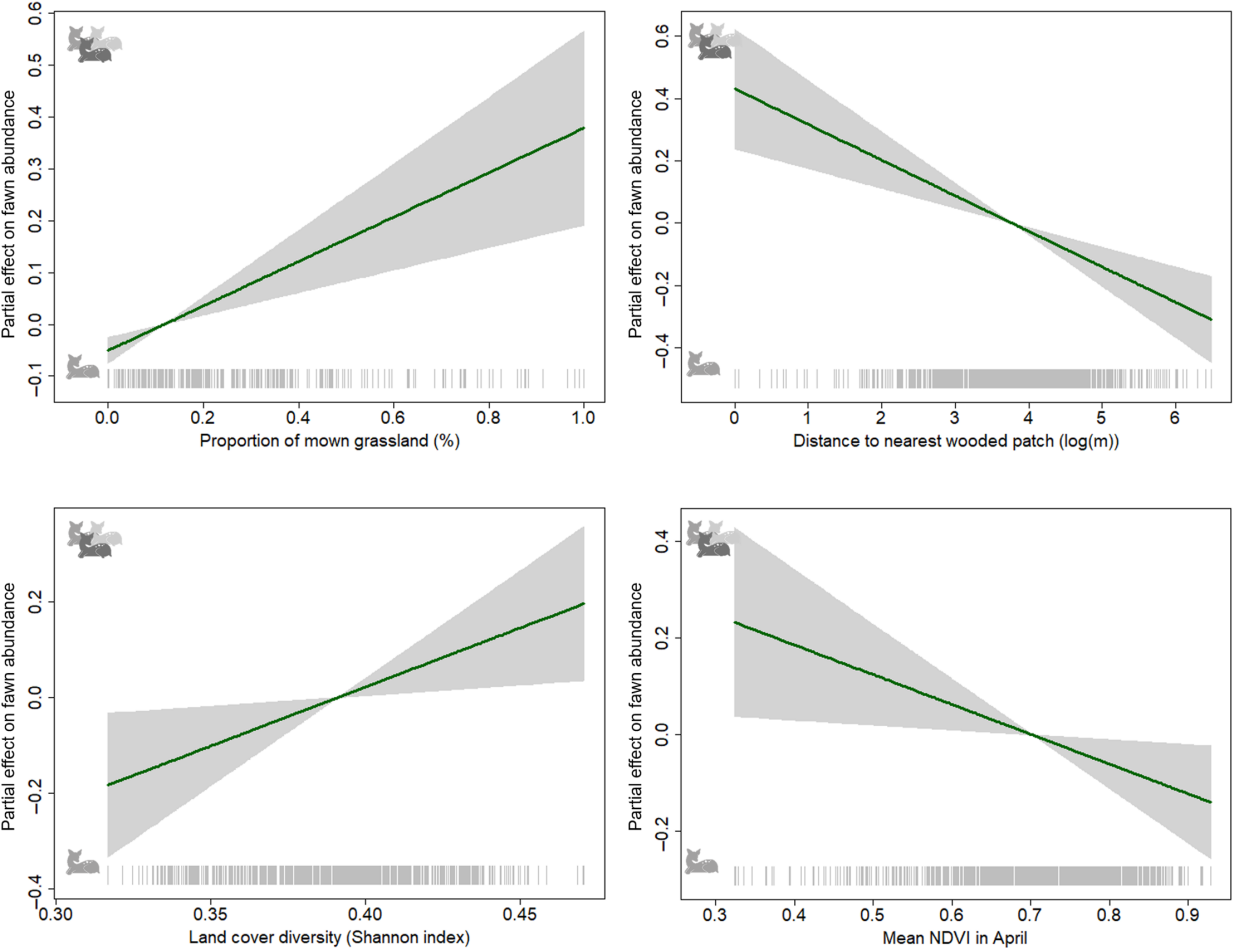
Partial regression plots for the significant explanatory variables (see Table 1) for the abundance of fawns in a field (*μ*). Shaded grey areas represent the standard errors. Positive values (multiple fawns) indicate a higher number of fawns being present.

The habitat descriptors influencing fawn abundance in a field, given that there was at least one fawn in that field, are modelled by the *μ*‐component of the GAMLSS. Here, a positive estimate indicates that an increase in the value of the descriptor increases fawn abundance. The abundance of fawns increased significantly with the proportion of meadows surrounding the field of interest that had already been mown [0.429 ± 0.116 (*Estimate* ± SE)]. Likewise, the abundance of fawns increased significantly as the distance to wooded patches decreased [−0.114 ± 0.026 (*Estimate* ± SE)] and as land‐cover diversity increased [2.472 ± 0.997 (*Estimate* ± SE)]. Finally, fawn abundance increased as mean NDVI in April decreased [−0.615 ± 0.266 (*Estimate* ± SE); Table 1 and Figure 4].

### 3.2 Within‐field selection

At the within‐field scale, among the candidate models‐set to explain the probability of a fawn bed‐site, the model with the highest support (lowest AIC value) was also the most parsimonious (fewest parameters; Table 2) and contained the distance to wooded patches, distance to roads and fawn detectability. Eight other models were within ∆AICc_Model_ ≤ 2, which additionally contained vegetation height, SSI, age class and age class as interaction term (Table 2).

**TABLE 2:** Model results for the within‐field scale analysis bed‐site selection (clogit) presenting the most supported models within ∆ AICc_Model_ ≤ 2 units.

Model	AICc	∆AICc_Model_	AIC weight	*k*
Distance to wooded patch + Distance to road + Detectability for predators	291.17	0	0.19	3
Distance to wooded patch + Distance to road + Detectability for predators + Vegetation height	291.50	0.33	0.16	4
Distance to wooded patch + Distance to road + Detectability for predators + Age	291.89	0.72	0.14	4
Distance to wooded patch + Distance to road + Detectability for predators + Vegetation height + Age	292.26	1.09	0.11	5
Distance to wooded patch + Distance to road + Detectability for predators + Age + Distance to structure × Age	292.66	1.48	0.09	5
Distance to wooded patch + Distance to road + Detectability for predators + Age + Detectability for predators × Age	292.96	1.78	0.08	5
Distance to wooded patch + Distance to road + Detectability for predators + Vegetation height + Age + Distance to wooded patch × Age	293.08	1.90	0.08	6
Distance to wooded patch + Distance to road + Detectability for predators + Vegetation height + Age + Detectability for predators × Age	293.12	1.95	0.07	6
Distance to wooded patch + Distance to road + Detectability for predators + SSI	293.15	1.97	0.07	4

*Note*: The parameters shown for each model are AICc, ∆ AICc_Model_, AICweight and *k* (number of estimable model parameters).

The best model, however, included a significant preference for bed‐sites with low detectability [−0.006 ± 0.001 (*β* ± SE)]. Fawns preferred areas further from wooded patches [0.295 ± 0.138 (*β* ± SE)] and further from roads [0.679 ± 0.278 (*β* ± SE)]. In contrast to the among‐field scale, vegetation height was not included in the best model, and the SSI as well as the age classification, neither as single term nor as interaction, significantly influenced bed‐site selection at the finer scale (Table 3).

**TABLE 3:** Model averaged beta coefficients, standard errors (SE), *z*‐values and their significance for finer scale bed‐site selection (*n* = 322/267) of roe deer fawns in agricultural areas.

Variable	Beta	SE	*z*‐Value	Pr(>|z|)	Signif. codes
Detectability for predators	−0.0063	0.0013	−4.901	<0.001	***
Distance to wooded patch	0.2951	0.1384	2.132	0.0330	*
Distance to road	0.6785	0.2783	2.438	0.0148	*

*Note*: Signif. codes: ***p < 0; **.001; *.01; ‘.’.05.

## 4 DISCUSSION

By analysing both among‐ and within‐field scales, we essentially differentiated between the maternal and neonatal behavioural components of bed‐site selection of roe deer fawns in the context of spring mowing. At the among‐field scale, higher fawn densities in fields where the surroundings have already been mown indicate strong direct effects of farming activities on bed‐site selection. Landscape heterogeneity was the most important descriptor driving preferential use of locations with access to both foraging habitats and refuge habitats. The selection for closer proximities to roads showed that, in this case, human disturbance seemed to be less influential. At the within‐field scale, besides thermoregulation, proxies for predator avoidance were most important, including bed‐sites further from roads. In some aspects, the equal and sometimes contrasting results of both scales reflect the same underlying driver: the high safety requirements of the female for herself and her fawn at the coarser scale and of the fawn at the finer scale.

### 4.1 Among‐field bed‐site selection

At the among‐field scale, we modelled fawn presence and abundance in relation to habitat characteristics which mostly reflect maternal behavioural decisions on where to give birth and care for her young within her home range rather than the behaviour of the fawn itself. We assume that selection for suitable habitat for giving birth and rearing are the underlying drivers of female behaviour during this time.

#### 4.1.1 Forage availability

With respect to H1a, we predicted that fawns would be more likely to be present and/or are more abundant in areas with good foraging conditions. Surprisingly, we found no significant effect of cropland and forage quality (i.e. NDVI) on fawn presence or abundance. Instead, lower NDVI values in April led to higher fawn abundance. Although other studies have demonstrated the importance of forage for bed‐site selection in other species (Barbknecht et al., 2011; Kjellander et al., 2012, *Dama dama*; Rearden et al., 2011, *Cervus elaphus nelsoni*), we suggest that the generally high productivity of our study region might decrease the relevance of such indices (Pettorelli et al., 2006). Alternatively, this result could hint at the general need of the female to trade the productivity of an environment with predator avoidance and to give priority to the safety of the fawn (Ciuti et al., 2006). Cropland may provide rich food resources, but not necessarily sufficient cover, particularly at the beginning of the mowing season. Towards the end of the mowing season, when croplands additionally provide sufficient cover, most other habitat types offer equally suitable conditions (Linnell et al., 2004), making cropland less attractive.

Next, as expected under H1a, the abundance of fawns was higher when surrounding fields had already been mown. This result indicates that, due to the habitat loss from mowing, females relocated their offspring to a field which provided sufficient vegetation for both foraging and concealment, while tolerating higher fawn densities. This is concordant with the findings of Linnell et al. (2004) who showed that the habitat use of fawns in agricultural landscapes reflects phenological changes in cover.

#### 4.1.2 Landscape heterogeneity

Besides high forage quality, females might also prefer to give birth and care for their young in fields that are part of a diverse local landscape. Indeed, we found that land‐cover diversity was a reliable predictor for fawn abundance and for fawn presence (H1b). This could substitute for the low importance of NDVI.

This finding is consistent with research showing that fragmented and diverse agricultural landscapes with field‐forest edges provide high‐quality resources (Hewison et al., 2009; Panzacchi et al., 2009). In fact, we also found that females placed their fawns in locations where there were forests and hedges nearby (H1b). Van Moorter et al. (2009) found increased fawn survival in regions with high edge density. Thus, heterogeneous landscapes appear to fulfil two important requirements for the doe: adequate foraging possibilities and sufficient concealment (Christen et al., 2018; McLoughlin et al., 2007; Tufto et al., 1996).

#### 4.1.3 Anthropogenic risk

In agreement with our prediction (H1c), at the broader among‐field scale, females placed their fawns far from man‐made structures, possibly due to higher perceived risk by free‐roaming dogs, or due to the disturbance created by human activity. Interestingly, females did not seem to be disturbed in the same way by roads, as fawns were found more often in fields with roads nearby (presence/absence). Although other studies indicated that roads are a source of disturbance at other times of the year (e.g. Bonnot et al., 2013), we speculate that this discrepancy may result from the different spatial scales of the behavioural response of the mother to risk‐avoidance and forage selection (Rearden et al., 2011). For example, at a comparative scale to ours, Berger (2007) found that moose (*Alces alces*) deliberately seek out paved‐roads for parturition as a shield against streets‐averse brown bears (*Ursus arctos*). Additionally, the lower availability of habitat patches far away from roads in our rather heterogeneous landscape may also prevent roe deer avoiding roads while accounting for other, possibly more important, resource needs. Lastly, there might be other not considered landscape features confounding our results.

### 4.2 Within‐field bed‐site selection

#### 4.2.1 Concealment against predation and hypothermia

Preference for high concealment at bed‐sites was previously shown in studies for other ungulates (*Odocoileus virginianus*, *O. hemionus*, Gerlach & Vaughan, 1991; Grovenburg et al., 2010). As predicted, our results indicate that fawns prefer bed‐sites with low detectability by predators (H2b), as, for example Panzacchi et al. (2009) suggested for roe deer and Kjellander et al. (2012) suggested for fallow deer. In contrast to our expectations and similar to Michel et al. (2020) for white‐tailed deer (*Odocoileus virginianus*), we did not find that vegetation height significantly differed between bed‐sites and random sites. Therefore, we assume that fawns have chosen their bed‐site in relation to vegetation density rather than height.

Interestingly, at this finer spatial scale, fawns preferred longer distances to roads compared to paired random sites, and this is in accordance with our prediction. In addition, and contrary to our expectations, fawns preferred bed‐sites that were further away from wooded patches (H2a). Fawns possibly unknowingly benefit from locations further away from edges, as predators such as red foxes (*Vulpes vulpes*) strategically observe forest edges (Jarnemo, 2004). Moreover, since females defend their fawns against foxes, higher distances to habitat edges could give the female time to spot and chase the fox away. This highlights the high security demands of the fawns, presumably as they are less mobile and more vulnerable to disturbance and predation. These findings are in line with other studies where predation was also the major factor in site selection at the finer scale (Barbknecht et al., 2011; Rearden et al., 2011).

With respect to thermoregulation, we did not find any impact of our measure of thermal conditions on fawn bed‐site selection (H2b). In contrast, mule deer (*Odocoileus hemionus*) fawns showed a clear preference for south‐exposed bed‐sites in an altitudinal diverse habitat in Colorado, USA (Gerlach & Vaughan, 1991) as did roe deer fawns in Switzerland, especially, in low‐density populations (Kurt, 1968). Huegel et al. (1986) reported an association between cool days and selection for bed‐sites on slopes providing maximum solar radiation for white‐tailed deer (*Odocoileus virginianus*). We believe that the lack of a similar relationship in our data could be the result of the rather homogeneous nature of the available habitat in terms of elevation and climatic conditions.

Finally, our metric of thermal conditions is likely not sufficiently sensitive to index the temperature and ground‐level climate of a bed‐site. Indeed, the height and density of the vegetation are also critical. Grovenburg et al. (2010) suggested that optimal body temperatures could rather be achieved in cooler regions when vegetation density is low due to direct sun exposure. Overall, predation avoidance seems to be the most critical factor driving the choice of bed‐site selection by fawns, even though beneficial thermoregulation conditions could potentially be indicated by the preference for dense vegetation.

## 5 CONCLUSION

In summary, our findings provide invaluable information for farmers and wildlife managers to target fawn rescue operations during mowing activities. We believe that our results are transferable to other regions due to our large‐scale study design in terms of our sample size (*n* > 600 fawn bed‐sites) and the geographic coverage, which is representative of a typical rural landscape in much of the species' continental range. These findings could help to prioritise areas that are potentially preferred by roe deer fawns as bed‐sites. In particular, such prioritisation of efforts can be essential because of a lack of manpower and because the flight time for UAV searches is commonly constrained by the battery charge. Further, the thermal difference between meadow and fawn is strongest in the early morning hours and decreases as solar radiation warms the meadow after dawn. Our work indicates that wildlife managers should focus their searches on fields that occur in more diverse and rural local landscapes, with nearby wooded patches, and particularly if many surrounding fields have already been mown. In addition, specific attention should be paid to those parts of the field with particularly dense vegetation, likely far from the woodland edge or road, particularly as hidden fawns in dense vegetation are hard to detect. These results can be used in combination with knowledge about the phenology of birth events (Kauffert et al., 2023) and technical solutions (detection and deterrence measures) to allocate time in a targeted manner to appropriate rescue measures and thus reduce the probability of fawns being mowed.

## AUTHOR CONTRIBUTIONS

**Sophie Baur:** Conceptualization (equal); data curation (lead); formal analysis (lead); investigation (lead); methodology (lead); visualization (lead); writing – original draft (lead); writing – review and editing (lead). **Johanna Kauffert:** Conceptualization (equal); data curation (lead); formal analysis (lead); investigation (lead); methodology (lead); visualization (lead); writing – original draft (lead); writing – review and editing (lead). **A. J. Mark Hewison:** Conceptualization (equal); methodology (equal); writing – review and editing (equal). **Sophie Reinermann:** Formal analysis (supporting); methodology (supporting); writing – review and editing (supporting). **Andreas König:** Funding acquisition (equal); project administration (equal); writing – review and editing (supporting). **Annette Menzel:** Conceptualization (equal); funding acquisition (equal); project administration (equal); supervision (equal); writing – review and editing (equal). **Wibke Peters:** Conceptualization (equal); funding acquisition (equal); project administration (equal); supervision (equal); writing – review and editing (equal).

## FUNDING INFORMATION

This research was funded by the Bavarian State Ministry of Food, Agriculture, and Forestry, grant number: A/19/17. Open Access funding enabled and organized by Projekt DEAL.

## CONFLICT OF INTEREST STATEMENT

None declared.

## Supporting information


Data S1.

## Data Availability

Data is permanently archived on Figshare (https://doi.org/10.6084/m9.figshare.24440572). Exact fawn locations cannot be published due to reasons of privacy rights of landowners.
