# Supplementary material for: Spatial scaling in bed‐site selection by roe deer fawns: Implications for mitigating neonatal mortality during mowing
Source: Ecol Evol. 2023 Nov 28;13(11):e10729. doi: 10.1002/ece3.10729 (PMC10682894; doi:10.1002/ece3.10729)
Supplement: Supplementary file 1 — Data S1. [file ECE3-13-e10729-s001.docx]

# Appendix

## AIC selection among-field selection

Table A1: AIC values of univariate models of fawn presence (vs. absence, i.e. fawn-free fields) and abundance in relation to habitat characteristics measured within 100 and 200 m buffer radii around the fawn bed site. Generalized Additive Models for Location, Scale, and Shape (GAMLSS) were fitted separately for each habitat descriptor and radius with a zero-adjusted Gamma distribution (ZAGA).

| Variable | 100 m | 200 m |
| --- | --- | --- |
| Mean NDVI close to day of search | 884.79 | 885.30 |
| SD NDVI close to day of search | 878.69 | 883.21 |
| Min NDVI close to day of search | 878.42 | 883.25 |
| Max NDVI close to day of search | 886.44 | 886.02 |
| Mean NDVI in April | 858.79 | 859.94 |
| SD NDVI in April | 868.42 | 876.00 |
| Min NDVI in April | 867.03 | 882.34 |
| Max NDVI in April | 882.16 | 881.45 |
| Proportion of forest | 865.72 | 863.72 |
| Proportion of grassland | 876.21 | 874.54 |
| Proportion of crops | 872.15 | 873.51 |
| Proportion of man-made structures | 878.30 | 881.82 |
| Number of land-use classes | 876.49 | 882.50 |
| Number of land-use patches | 876.63 | 879.13 |
| Shannon Index | 872.41 | 873.90 |
| Edge density | 867.60 | 869.91 |
| Edge length | 867.60 | 869.90 |
